# Supervised versus non-supervised implementation of an oral health care guideline in (residential) care homes: a cluster randomized controlled clinical trial

**DOI:** 10.1186/1472-6831-10-17

**Published:** 2010-07-02

**Authors:** Gert-Jan van der Putten, Luc De Visschere, Jos Schols, Cees de Baat, Jacques Vanobbergen

**Affiliations:** 1 Birkhoven Care-estate, P.O. Box 363, 3800 AJ Amersfoort, The Netherlands; 2 Department of Community Dentistry and Oral Public Health, Ghent University, Belgium; 3 Department of General Practice, Section Nursing Home Medicine, CAPHRI, Maastricht University, The Netherlands; 4 Department of Oral Function and Prosthetic Dentistry, Radboud University Nijmegen Medical Centre, Nijmegen, The Netherlands

## Abstract

**Background:**

The increase of the proportion of elderly people has implications for health care services. Advances in oral health care and treatment have resulted in a reduced number of edentulous individuals. An increasing number of dentate elderly people have tooth wear, periodontal disease, oral implants, and sophisticated restorations and prostheses. Hence, they are in need of both preventive and curative oral health care continuously. Weakened oral health due to neglect of self care and professional care and due to reduced oral health care utilization is already present when elderly people are still community-dwelling. At the moment of (residential) care home admittance, many elderly people are in need of oral health care urgently. The key factor in realizing and maintaining good oral health is daily oral hygiene care. For proper daily oral hygiene care, many residents are dependent on nurses and nurse aides. In 2007, the Dutch guideline "Oral health care in (residential) care homes for elderly people" was developed. Previous implementation research studies have revealed that implementation of a guideline is very complicated. The overall aim of this study is to compare a supervised versus a non-supervised implementation of the guideline in The Netherlands and Flanders (Belgium).

**Methods/Design:**

The study is a cluster randomized intervention trial with an institution as unit of randomization. A random sample of 12 (residential) care homes accommodating somatic as well as psycho-geriatric residents in The Netherlands as well as in Flanders (Belgium) are randomly allocated to an intervention or control group. Representative samples of 30 residents in each of the 24 (residential) care homes are monitored during a 6-months period. The intervention consists of supervised implementation of the guideline and a daily oral health care protocol. Primary outcome variable is the oral hygiene level of the participating residents. To determine the stimulating or inhibiting factors of the implementation project and the nurses' and nurse aides' compliance and perceived barriers, a process evaluation is carried out.

**Discussion:**

The method of cluster randomization may result in a random effect and cluster selection bias, which has to be taken into account when analyzing and interpreting the results.

**Trial registration:**

Current Controlled Trials ISRCTN86156614

## Background

The international literature shows that the proportion of elderly people has increased considerably during the last decades and is expected to further increase during the next decades. This demographic shift will have important implications for health care services. More (frail) elderly people will present more morbidity and care dependency and, consequently, will need an increasing proportion of health care services [1]. Those elderly people, who are not able to function independently, are often supported by domiciliary care service or admitted to (residential) care homes [2,3].

Advances in oral health care and treatment during the last decades have resulted in a reduced number of edentulous individuals. A still increasing number of dentate elderly people have tooth wear, periodontal disease, oral implants, and sophisticated tooth- and implant-supported restorations and prostheses. Hence, they are in need of both preventive and curative oral health care continuously. Complexity of the oral conditions, oral mucosal lesions, systemic diseases, and medication use make (frail) elderly people more vulnerable to oral problems than younger age groups, especially when they are cognitively impaired [4,5]. Weakened oral health due to neglect of self care and professional care and due to reduced oral health care utilization is already present when (cognitively impaired) elderly people are still community-dwelling [5-8]. At the moment of (residential) care home admittance, many elderly people in countries all over the world are in need of oral health care urgently. If their needs are not met, their oral health will be persistently poor and will utmost probably further deteriorate during their residency because of increasing care dependency and subsequent lack of adequate oral health care [9-14].

Systemic diseases affect oral health and vice versa [15,16]. Several medications have also a negative effect on oral health by inducing xerostomia, hyposalivation, mucosal lesions, and abnormal bleeding [17]. Hyposalivation is a specific problem because saliva plays a major role in protecting both hard and soft oral tissues [18]. Furthermore, several aspects of oral health are affecting quality of life and well-being [19-21]. Oral health influences mastication, food selection, weight, speech, taste, hydration, appearance, and psycho-social behaviour and is thereby a concern not only for the elderly individuals themselves, but also for their relatives and care providers [22-25].

The key factor in realizing and maintaining good oral health is daily oral hygiene care by removing the oral bacterial plaque, mainly composed of pathogenic gram-negative germs [26,27]. However, many residents of residential care homes and long-term care facilities are not able to clean their mouths and eventually removable dentures themselves. For proper daily oral hygiene care, they are dependent on nurses and nurse aides [28,29]. However, the importance of oral health of residents is often misunderstood and neglected by nurses and nurse aides [30]. A lack of oral health knowledge and oral health care skills of even qualified nurses is an important inhibiting factor in achieving an acceptable level of residents' oral hygiene [31]. No prioritisation to oral health care of the residents themselves and their family is another barrier of proper oral health and daily oral hygiene care [32,33]. Furthermore, in many cases a resident's repeated resistiveness is disincentive for nurses and nurse aides, leading to inadequate daily oral hygiene care [34]. Teaching and qualifying nurses and nurse aides in providing individual oral health care for residents had until recently a low priority in managers and physicians of residential care homes and long-term care facilities [35,36]. Convincing the managers and physicians of the benefits of oral health and adequate oral health care as well as improving the oral health knowledge and oral health care attitude and skills of nurses and nurse aides may contribute to an improvement of oral health and quality of life of residents. Although during the last several years increasing attention has been paid to improving oral health care, there is still a need for guidelines and effective protocols, for oral health and oral hygiene assessment tools for nurses and nurse aides, and for teaching nurses and nurse aides practical skills of daily oral hygiene care [37-39]. In 2007, the Dutch guideline *"Oral health care in (residential) care homes for elderly people" *was developed and presented to all (residential) care homes for elderly people in The Netherlands and a part of Flanders, Belgium. The Dutch guideline is satisfying the Appraisal of Guidelines Research & Evaluation Instrument (AGREE) [40]. It describes all aspects of good oral health and oral health care, presents the methods and skills needed for providing oral health care to residents, and presents effective oral health and oral hygiene assessment tools. The ultimate objective of the guideline is to improve the oral health of the residents.

Any care guideline needs careful implementation as well as research for assessing its residents' and care providers' compliance. Guideline implementation involves the concrete activities and interventions undertaken to turn policies into desired results. Previous implementation research studies have revealed that implementation of a guideline is very complicated. Although numerous attempts have been made, an effective implementation method has not yet been discovered. Key factors are 'buying in' the care providers, determining during the implementation project which factors are stimulating or inhibiting the project, and determining the care providers' perceived barriers and compliance [41-48].

### Scientific hypothesis

The scientific hypothesis of the present study is that supervised implementation of the guideline "*Oral health care in (residential) care homes for elderly people*" is more effective in improving oral health and oral health care of the residents when compared to non-supervised implementation.

### Aim and objectives

The overall aim of the study is to compare a supervised versus a non-supervised implementation of the guideline "*Oral health care in (residential) care homes for elderly people"*. The aim can be rendered into 5 research questions:

1. Is there any statistically significant difference between oral hygiene levels of elderly residents in (residential) care homes with supervised implementation of the guideline when compared to those in (residential) care homes without supervised implementation of the guideline?

2. Is there at care home level any statistically significant difference between attitude and knowledge level of nurses and nurse aides of (residential) care homes with supervised implementation of the guideline when compared to those in (residential) care homes without supervised implementation of the guideline?

3. Is there any statistically significant difference in impact on the outcome variables of research questions 1 and 2 between the (residential) care homes in The Netherlands when compared to Flanders (Belgium) and which factors are causing the country differences?

4. Which factors are stimulating or inhibiting the implementation of the guideline in the (residential) care homes in The Netherlands and Flanders (Belgium)?

5. What is the compliance of and which barriers are perceived by the nurses and nurse aides in (residential) care homes in The Netherlands and Flanders (Belgium) while implementing the guideline?

## Methods/Design

### Design of the study

The study is a cluster randomized intervention trial with an institution as the unit of randomization. A random sample of 12 (residential) care homes accommodating a total of 120-180 somatic as well as psycho-geriatric residents in The Netherlands as well as in Flanders (Belgium) are randomly allocated to an intervention or control group. Representative samples of 30 residents in each of the 24 (residential) care homes are monitored during a 6-months period. Research data are gathered at baseline and at 6 months after the start of the study. The study is supervised and monitored by 2 investigators, the first and second author of this article. In each institution, an institution study supervisor, appointed by the managing director, is responsible for executing all study activities. The intervention consists of supervised implementation of the guideline "*Oral health care (residential) care homes for elderly people*" and a daily oral health care protocol. This protocol is derived from the guideline and formulated by the authors. Primary outcome variable is the oral hygiene level of the participating residents. To determine the stimulating or inhibiting factors of the implementation project and the nurses' and nurse aides' compliance and perceived barriers, a process evaluation is carried out [49]. The study is conducted according to the principles of the Declaration of Helsinki (version 17c, 2004) and in accordance with the Medical Research Involving Human Subjects Act (WMO). The study protocol is approved by the Ethics Committees of the Ghent University, Belgium (OG017 - approval 2008/440) and the Radboud University Nijmegen, The Netherlands (NL24666.091.08 approval 2008/273).

### Participants and setting

In each country, a sample of (residential) care homes for elderly people is obtained using stratified (geographical distribution) cluster sampling with replacement. A (residential) care home is considered eligible for inclusion unless any of the following exclusion criteria are applicable:

• The management of the institution does not agree with the random allocation to the intervention or control group;

• The institution has mainly wards accommodating less than 20 residents;

• The institution has only somatic or psycho-geriatric wards;

• An oral health care guideline or protocol has already been introduced and implemented;

• Nurses and nurse aides have received special training on oral health care during the last 24 months;

• More than 5 other major care innovation projects have been implemented during the last 24 months.

Once the managing director of an institution agrees to participate by written informed consent, the institution is randomly allocated to either the intervention group or the control group of the country. Based on the power calculation (see following indentation) a representative cohort of 30 residents of the institution needs to be included.

Crucial factors for calculating the sample size (n) are the presumed distribution of the outcome measure (oral hygiene level) in the population of residents (*σ*), the presumed effect of the intervention (*μ*^1 ^- *μ*^2^), the power required (1-*β*), the a priori determined level of significance (*α*), and the value of the intraclass correlation (design effect). An a priori power of 80% and a level of significance of 0.05 are predetermined. The design effect (Deff) represents the ratio of the number of residents required using cluster randomization to the number of residents required using individual randomization.

The primary formula for calculating the sample size is: .

Deff = 1 + [(m - 1) *ρ*]; m = the number of residents in each institution, *ρ *= the intraclass correlation coefficient.

The adjusted sample size (n^adj^) equals: n × Deff. The number of residents in each institution (m) = n^adj ^: k; k = the number of institutions in the sample.

Based on previous studies, an intraclass correlation coefficient of 0.95 and a standard deviation (sd) for dental plaque and denture plaque of 0.75 and 0.88 respectively is used. A 25% improvement of oral hygiene level is the presumed effect of the intervention. With regard to the design effect and drop-outs, loss to follow up, and uncertainty in power calculation, a sample size of 360 residents and 12 clusters per country seems an achievable number for the 6-months period. This means 30 residents per (residential) care home.

To participate in the study, a resident should:

• Supply a written informed consent, undersigned by himself or his legal representative

• Have teeth and/or partial or complete dentures

• Have the cognitive and physical condition required for undergoing an oral examination

• Be residing in the institution during the entire 6-months period presumably.

Residents are excluded when in day-care, in short-term residency, in coma, in palliative care or terminally ill, using a denture adhesive, expressing verbal or physical resistiveness before or during an oral examination.

### Intervention

The intervention consists of supervised implementation of the guideline "*Oral health care in (residential) care homes for elderly people*" and the daily oral health care protocol derived from the guideline. In each institution of the intervention group, every ward head appoints a nurse who will act as ward oral health care organizer (WOO). The managing director, physician(s), ward heads, WOO's, nurses, nurse aides, and a sample of 30 residents are involved in the study. The implementation of the guideline is supervised by a dental hygienist and includes:

• A 1.5-hour informative oral presentation on the guideline, the daily oral health care protocol, and the supervised implementation project before the start of the study, introduced by the dental hygienist and one of the investigators and addressing the managing director, the institution study supervisor, the ward heads, and the WOO's. Important objective of the informative oral presentation is to lay a strong institutional foundation of the implementation project and the study.

• A 2-hour lecture and in total 3 hours of practical education for the WOO's. The education, presented by the dental hygienist, regards the theoretical and practical essentials of the guideline. The WOO's are practically educated in skills facilitating them to practically educate and encourage the nurses and nurse aides of theirs wards.

• A 1.5-hour theoretical and executive education session at each ward, presented by the WOO, for all ward nurses and nurse aides. This education session is scheduled after the baseline data collection. A summary of the guideline is presented and all executive actions, such as tooth brushing, are taught and demonstrated with ward residents on site. As from the education session, the WOO will encourage and assist the nurses and nurse aides regularly in the daily delivery of oral health care.

• Providing oral health care materials and products for each resident by the dental hygienist.

• Monitoring visits of the dental hygienist and an investigator every 6 weeks, meeting the institution study supervisor and WOO's for listing and resolving implementation and study problems.

### Data collection

**Figure 1:**
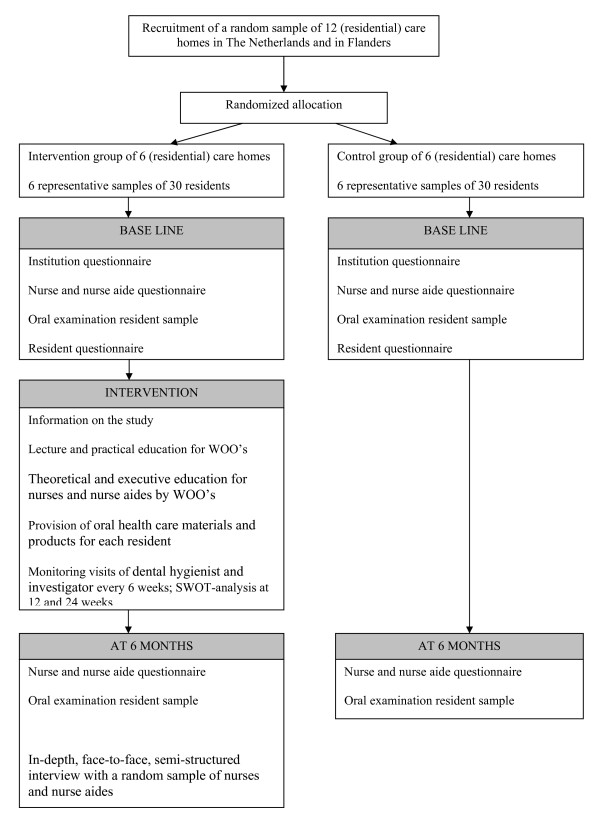
**Flowchart of the study protocol**.

Research data are gathered in the institutions of the intervention and the control group. At baseline, a questionnaire on the resident capacity of the institution, the mean length of stay of the entire group of residents, mean age of the entire group of residents, and the presence of oral health care providers is completed by the managing director of each institution. At baseline and at 6 months, an oral examination of the random sample of 30 residents is carried out by a team of trained external examiners (dentists, master dental students, and master dental hygiene students). They will carry out the data collection after exercising and calibrating the examination criteria and after determining their intra- and inter-examiners' reliability in a pilot study. The examiners do not know whether an institution is allocated to the intervention or the control group. At baseline, a questionnaire on personal and medical details of every resident of the random sample is completed. Furthermore, at baseline and at 6 months a questionnaire addressing the nurses and nurse aides is completed. Finally, at the end of the study, a process evaluation is conducted in the institutions of the intervention group to acquire insight in the stimulating and inhibiting factors of the implementation process. Figure 1 presents a flowchart of the study protocol and Table 1 presents an overview of the data collection.

**Table 1:** Overview of data collection

Data	Collection time	Purpose
**Institution questionnaire ** resident capacity mean length of stay of residents mean age of residents presence of oral health care providers	baseline baseline baseline baseline	comparison institutions comparison institutions comparison institutions comparison institutions

**Nurses and nurse aides questionnaire** gender age years of experience oral health knowledge attitude to personal oral health care	baseline/6 months baseline/6 months baseline/6 months baseline/6 months baseline/6 months	comparison nurses and nurse aides comparison nurses and nurse aides comparison nurses and nurse aides intervention effect intervention effect

**Resident questionnaire**		
Age	baseline	comparison residents
Gender	baseline	comparison residents
Primary diagnoses	baseline	comparison residents
secondary diagnoses	baseline	comparison residents
prescribed drugs	baseline	comparison residents
Care Dependency Scale	baseline	comparison residents
Mini Mental State Examination	baseline	comparison residents

**Oral examination** Plaque index (natural teeth, denture)	baseline/6 months	intervention effect

#### Oral examination

The oral hygiene level of natural teeth is assessed using the validated plaque index described by Silness and Löe (score range 0-3) at a subset of the so-called 'Ramfjörd teeth' [50]. In absence of one of these teeth, the corresponding distal neighbour tooth will be assessed. The oral hygiene level of dentures is assessed using a Methylene Blue^® ^denture plaque disclosing solution according to the method of Augsburger and Elahi (score range 0-4) [51].

#### Resident questionnaire

The resident questionnaire is completed by a physician of the institution and records personal details, primary diagnosis, secondary diagnoses, Care Dependency Scale (CDS) score [52], Mini Mental State Examination (MMSE) score [53], and prescribed drugs.

#### Nurse and nurse aide questionnaire

The self-administered validated questionnaire addressing the nurses and nurse aides covers personal details, years of experience, oral health knowledge, and attitude to personal oral health care.

#### Process evaluation

During monitoring visits of the dental hygienist and an investigator every 6 weeks during the intervention, meeting the institution study supervisor and WOO's, problems of the implementation project and the study are listed and resolved. At 12 and 24 weeks, a SWOT analysis is performed. SWOT is a strategic planning method for evaluating the Strengths, Weaknesses, Opportunities and Threats, identifying the favourable and unfavourable factors while achieving the implementation project objectives. A 10 items questionnaire is used concerning the progress, the involvement of the residents, and the organizational aspects of the implementation project. At the end of the study, the implementation project is evaluated by an in-depth, face-to-face, semi-structured interview with a random sample of nurses and nurse aides of each institution. Main questions of the semi-structured interview are on observed alterations of the oral health care provided in the institution, causality of these alterations and difficulties experienced during the implementation process. Subsequently, an adaptation of 'reflective listening' is used, a counselling technique eliciting a thorough disclosure of thoughts and feelings [54]. The technique involves reflecting what the interviewer believes was said in order to verify or clarify the nurses' and nurse aides' statements. Using this technique, the nurses and nurse aides are also actively confronted with eventual inconsistencies in their answers and statements. The interviews take 20-30 minutes and are conducted individually by the two investigators. All interviews are taped and transcribed.

### Statistical analysis

Both categorical and continuous variables are initially analyzed using exploratory data analysis, employing a variety of mostly graphical techniques and techniques for testing the necessary assumptions. The institution is the unit of randomization and the residents are the units of analysis. Cluster effects are addressed in the analysis. Intraclass correlation will be calculated for each outcome variable as a measure of correlation among residents within the institutions as well as among institutions within each country. The effect of the implementation project at individual, institution and country level is summarized and analyzed in a multilevel comparative analysis. Three comparative dimensions are handled simultaneously: the resident effect, the institution effect, and the country effect. Therefore, a three-level structure is used with resident (level 1) and institution (level 2), nested in broader organization units on country level (level 3). Differences in primary outcomes at baseline and differences between the intervention and the control group as well as between countries at baseline and at 6 month are calculated. Covariates at individual level are the subjects of the resident questionnaire and the nurse and nurse aide questionnaire.

Responses to the semi-structured interview of the process evaluation are analyzed using coding techniques commonly utilized for qualitative research methods [55]. Recurrent themes in the responses are used to set up a framework.

All research data are analyzed using MANOVA. A multilevel regression analysis is performed to determine the most important predicting factors with respect to oral hygiene level.

## Discussion

A cluster randomized controlled trial allows for statistical analysis of the feasibility and effectiveness of an intervention on care provision. This trial provides both practical and methodological advantages for implementation studies, especially when the intervention requires policy or behavioural alterations and intends an effect at institution level [56]. Cluster randomization using institutions as the unit of randomization reduces contamination between groups of persons. It is easier to deliver an intervention at institution level (unit) than at individual level within an institution. Also, when focussing on all nurses, nurse aides, and residents, group dynamics and peer pressure may facilitate the adoption of the intervention. On the other hand, cluster randomization may result in a random effect, which has to be taken into account when analyzing and interpreting the results. Another problem of cluster randomization is the hazard of selection bias at cluster level. An institution which, for one or another reason, decides to abandon the study may cause an important attrition bias. This is even of greater concern in case of drop outs of differential institutions in the intervention as well as in the control group. To prevent drop-out, all participating institutions are requested to provide a written informed consent for the entire study and study period.

The level of oral hygiene as primary outcome measure, a set of explanatory variables at different levels (resident, institution, and country), and the process evaluation data will allow revealing the supervised implementation effect and the stimulating and inhibiting factors.

An essential objective of the implementation project is to improve the oral health knowledge and the oral health care attitude and skills of the nurses and nurse aides. Many guidelines are, because of their lengthy and detailed character, rather difficult to access for nurses and nurse aides. The guideline used in this study provides also an easy to use daily oral health care protocol derived from the guideline, enabling the nurses and nurse aides to adhere the instructions and recommendations more easily.

## Competing interests

The authors declare that they have no competing interests.

## Authors' contributions

All authors participated in the conception and design of the study protocol. G-JvdP and LDV drafted the manuscript. JS, CdB and JV reviewed the draft manuscript. All authors read and approved the final manuscript.

## Pre-publication history

The pre-publication history for this paper can be accessed here:

http://www.biomedcentral.com/1472-6831/10/17/prepub
